# Isothermal Microcalorimetry Detects the Presence of Persister Cells in a *Staphylococcus aureus* Biofilm After Vancomycin Treatment

**DOI:** 10.3389/fmicb.2019.00332

**Published:** 2019-02-25

**Authors:** Maria Eugenia Butini, Gerardo Abbandonato, Carmine Di Rienzo, Andrej Trampuz, Mariagrazia Di Luca

**Affiliations:** ^1^Charité – Universitätsmedizin Berlin, Corporate Member of Freie Universität Berlin, Humboldt-Universität zu Berlin, and Berlin Institute of Health, Center for Musculoskeletal Surgery, Berlin, Germany; ^2^Berlin-Brandenburg Center for Regenerative Therapies, Charité – Universitätsmedizin Berlin, Berlin, Germany; ^3^NEST, Istituto Nanoscienze – Consiglio Nazionale delle Ricerche, Pisa, Italy; ^4^Center for Nanotechnology Innovation @ NEST, Istituto Italiano di Tecnologia, Pisa, Italy; ^5^Department of Biology, University of Pisa, Pisa, Italy

**Keywords:** *Staphylococcus aureus*, biofilm, persister cells, antibiotic activity, isothermal microcalorimetry, vancomycin, biofilm-associated infection

## Abstract

*Staphylococcus aureus* biofilm plays a major role in implant-associated infections. Here, the susceptibility of biofilm *S. aureus* to daptomycin, fosfomycin, vancomycin, trimethoprim/sulfamethoxazole, linezolid, and rifampicin was investigated by isothermal microcalorimetry (IMC). Moreover, the persister status of cells isolated from *S. aureus* biofilm after treatment with vancomycin was also analyzed. *S. aureus* biofilm was tolerant to all the antibiotics tested [minimum biofilm bactericidal concentration (MBBC) > 256 μg/ml], except to daptomycin [MBBC and minimum biofilm eradicating concentration (MBEC) = 32 μg/ml] and rifampin (MBBC and MBEC = 128 μg/ml). After the treatment of MRSA biofilm with 1024 μg/ml vancomycin, ∼5% cells survived, although metabolically inactive (persisters). Interestingly, IMC revealed that persister bacteria reverted to a normal-growing phenotype when inoculated into fresh medium without antibiotics. A staggered treatment of MRSA biofilm with vancomycin to kill all the metabolically active cells and daptomycin to kill persister cells eradicated the whole bacterial population. These results support the use in the clinical practice of a therapeutic regimen based on the use of two antibiotics to kill persister cells and eradicate MRSA biofilms. IMC represents a suitable technique to characterize in real-time the reversion from persister to metabolically-active cells.

## Introduction

*Staphylococcus aureus* is a pathogenic organism responsible for a wide variety of bacterial infections affecting different anatomic districts, including bloodstream, respiratory tract, skin, soft tissues, and bone (Kuroda et al., 2001; DeLeo and Chambers, 2009; Tong et al., 2015). *S. aureus* also colonizes necrotic tissues and abiotic surfaces, thus forming biofilms (Arciola et al., 2015). Within a biofilm, bacteria, embedded in an extracellular matrix, develop into a complex community presenting functional and structural heterogeneity (Zimmerli et al., 2004). *In vivo*, the biofilm extracellular matrix might protect the embedded bacterial cells from the attack of some antimicrobials and host immunity (Bjarnsholt et al., 2013).

Biofilms are also characterized by cells with a slow- and non-growing phenotype, which are able to survive in the presence of high concentrations of bactericidal antibiotics (Lewis, 2005), thus contributing to infection relapses. These so-called ‘persister cells’ represent a small percentage of a microbial population, which are genetically susceptible but phenotypically resistant to antimicrobials thanks to their non-growing quiescent state (Fisher et al., 2017; Grassi et al., 2017). Persistence is, therefore, markedly distinct from resistance, which is an inherited ability acquired by the total microbial population and it implies the capacity of bacteria to grow and replicate in the presence of high concentrations of antimicrobial with no dependency on the treatment duration (Scholar and Pratt, 2000; Brauner et al., 2016).

Even though persister cells may be stochastically induced (Maisonneuve et al., 2013), their emergence can also be fostered as a stress response to particular environmental conditions, such as high concentrations of antibiotics (Krut et al., 2004) and nutritional restrictions (Nguyen et al., 2011). Additionally, the amount of cells that spontaneously enters this dormant state depends on the growth stage of a culture (Wood et al., 2013). Indeed, levels of persisters frequently increase during the stationary growth phase and in biofilms, most likely functioning as bacterial survival strategy. In fact, when the antibiotic treatment kills normal-growing cells, persisters survive and resuscitate upon treatment interruption, thus repopulating the biofilm (Lewis, 2007).

The physical shielding offered by the extracellular matrix, the bacterial resistance (Brauner et al., 2016) and the ability of persister cells to survive lethal treatments represent some of the main causes of infection recalcitrance and therapeutic failure (Lewis, 2008; Conlon et al., 2016).

The increasing use in medicine of indwelling devices, such as orthopedic joint prosthesis, cardiac valves, breast implants and also catheters, fosters the development of infections difficult to treat due to bacteria (including *S. aureus*) forming biofilm on the material surface. Current available therapeutic options include drugs with bactericidal and bacteriostatic activities, both as monotherapy and in combination regimens, such as vancomycin, daptomycin, linezolid, trimethoprim/sulfamethoxazole, rifampicin, and fosfomycin. Vancomycin remains one of the pharmacological choices for the prophylaxis or treatment of invasive staphylococcal infections (Liu et al., 2011) and it is the antibiotic of first choice after other treatments failed (Boneca and Chiosis, 2003). This glycopeptide exerts a slow bactericidal activity against gram positive bacteria inhibiting the cell wall biosynthesis by strongly binding to the terminal D-Ala-D-Ala dipeptide on the nascent peptidoglycan chains (Katzung et al., 2012).

The aims of this study are: (i) to investigate the *in vitro* anti-biofilm activity of selected antibiotics commonly used in the clinical practice for the management of implant-associated infections due to methicillin- resistant and methicillin-susceptible *S. aureus* by conventional tests and isothermal microcalorimetry (IMC); (ii) to evaluate the presence of persister cells in biofilm grown *in vitro* after the exposure to high bactericidal concentrations of vancomycin; (iii) to characterize the persisters metabolic activity and their susceptibility to bactericidal antibiotic agents, such as daptomycin.

## Materials and Methods

### Staphylococcal Strains

Methicillin-resistant *S. aureus* (MRSA) ATCC 43300 and methicillin-susceptible *S. aureus* (MSSA) ATCC 29213 were used in this study. Bacteria were stored in cryovial bead preservation systems (Microbank; PRO-LAB DIAGNOSTICS, Richmond Hill, ON, Canada) at -80°C. All bacterial strains were cultured on Tryptic Soy Agar (TSA) (VWR Chemicals, Leuven, Belgium) for 24 h at 37°C in an ambient air incubator. Inocula were prepared according to a McFarland standard turbidity of 0.5 (λ = 565 ± 15 nm) and determined by colony forming units (CFUs) counting.

### Antimicrobial Agents

Fosfomycin (InfectoPharm, Heppenheim, Germany), rifampicin (Sandoz Pharmaceuticals, Steinhausen, Switzerland) and vancomycin (Hexal, Holzkirchen, Germany) were obtained as powder and reconstituted using sterile pyrogen-free water. Daptomycin (Novartis Pharma Schweiz, Basel, Switzerland) was obtained as a powder and dissolved in sterile pyrogen-free 0.9% saline. Trimethoprim/sulfamethoxazole (ratio 1:5) (Roche Pharma, Reinach, Schweiz) and linezolid (Pfizer Pharma, Berlin, Germany) were supplied in liquid form.

### Evaluation of the Minimum Inhibitory Concentration (MIC) by Broth Macrodilution and Etest

The MICs of different antibiotics against planktonic *S. aureus* cells were evaluated using either broth macrodilution according to the CLSI guidelines (CLSI, 2012) or Etest (bioMérieux SA, Lyon, France). Briefly, a standard inoculum of 1–5 10^5^ CFUs/ml was prepared in Cation Adjusted Müller Hinton broth (CAMHB) (Becton, Dickinson and Company, Le Pont-de-Claix, France) and serial twofold dilutions of each antibiotic were added to the test tubes. Next, macrodilution tubes were incubated at 37°C for 18 h. The MIC was defined as the lowest concentration of antimicrobial that completely inhibited the growth of the organism in the tubes, as detected by unaided eye. Etest was performed on Müller Hinton Agar (MHA) according to the manufacturer’s instructions. After incubation at 37°C for 18 h, the MIC was determined as the concentration value where the inhibition ellipse intersected the scale of the strip.

### Isothermal Microcalorimetry (IMC) Assay

Isothermal microcalorimetric analysis was performed as previously described (Butini et al., 2018b). Briefly, an isothermal microcalorimeter (TAM III; TA Instruments, New Castle, DE, United States) equipped with 48 calorimeter channels and a detection limit of 0.2 μW was used. Glass ampoules were sealed for air tightness and introduced into the microcalorimeter, first in the equilibration position and, after 15 min, in the measuring position. Heat flow (μW) and total heat (J) were monitored in real-time for 24 and 48 h when testing antibiotics against planktonic and biofilm bacteria, respectively.

### Evaluation of the Antibiotic Activity Against Planktonic Bacteria by IMC

For the microcalorimetric investigation of antibiotic activity against planktonic MRSA and MSSA, glass ampoules were inoculated with 1–5 × 10^5^ CFUs/ml cells and twofold serial dilutions of antibiotic in CAMHB. When daptomycin and fosfomycin, were tested, CAMHB was supplemented with 50 mg/l Ca^2+^ and 25 mg/l glucose-6-phosphate, respectively. Ampoules containing inoculated CAMHB without any antibiotics were included as growth control and ampoules with sterile CAMHB as negative control. The minimum heat inhibitory concentration (MHIC) for planktonic was defined as the lowest antimicrobial concentration that inhibited growth-related heat production during 24 h-incubation in the microcalorimeter, as previously reported (Oliva et al., 2014; Gonzalez Moreno et al., 2017; Butini et al., 2018b). For each antibiotic two independent experiments were performed, if the two MHIC values were discordant, a third experiment was carried out.

### Evaluation of the Anti-Biofilm Activity by IMC and Sonication/Colony Counting

Porous glass beads (VitraPor; ROBU, Hatter, Germany). Diameter, 4 mm; pore size, 60 μm; porosity, 0.2 m^2^/g) were statically incubated in Trypticase Soya broth (TSB) inoculated with 2–3 bacterial colonies (corresponding to ∼1 × 10^7^ CFUs/ml) for 24 h at 37°C. The ratio between glass beads and bacterial suspension (ml) was 1:1. After incubation, beads were washed three times with 0.9% sterile saline and exposed to twofold serial dilutions of antibiotic in CAMHB for 24 h. A positive control consisting in biofilm incubated without treatment was included, as well as a sterile bead as a negative control. Next, beads were rinsed (3x) and placed in glass ampoules containing 3 ml of fresh CAMHB. The minimum biofilm bactericidal concentration (MBBC) was defined as the lowest antimicrobial concentration that strongly reduced the number of viable bacterial cells within the biofilm, therefore leading to undetectable heat values (no biofilm cell metabolic activity) during 48 h-incubation in fresh medium (Butini et al., 2018b). For each antibiotic two independent experiments were performed, if the two MBBC values were discordant, a third experiment was carried out.

The biofilm beads treated with MBBC of each antibiotic were collected and further analyzed by sonication and colony counting. Briefly, after calorimetric analysis, beads were washed and transferred to a 2 ml Eppendorf tubes containing 1 ml PBS (pH 7.4, 10 mM), vortexed for 30 s, sonicated at 40 kHz for 1 min in a bath sonication instrument at 40 kHz and 0.2 W/cm^2^ (BactoSonic, Bandelin, Berlin, Germany) and finally vortexed for 30 s, as previously described (Trampuz et al., 2007). The sonication fluid was serially diluted and aliquots thereof were plated on TSA (plate diameter 92 mm) and incubated at 37 °C for 24 h. Bacterial colonies were expressed as CFUs/ml (plating detection limit = 20 CFUs/ml). The minimum biofilm eradicating concentration (MBEC) was then defined as the lowest antibiotic concentration required to kill all sessile cells (0 CFUs/bead on plate counts) (Butini et al., 2018b).

The pharmacodynamic parameters (MBBC and MBEC) used to quantify the anti-biofilm activity of antibiotics measured by IMC were defined according to the suggestions reported by Macia et al. (2014).

### Isolation and Characterization of Persister Cells From Biofilms After Treatment With High Concentrations of Vancomycin

To isolate persister cells of MRSA, a biofilm, formed as previously described, was exposed to 1024 μg/ml vancomycin. Beads with untreated biofilm were included as growth control, as well as sterile beads as negative control. Next, beads were washed and sonicated as described above to dislodge cells survived to the antibiotic treatment. Bacteria were diluted to a final concentration of ∼1 × 10^5^ CFUs/ml in PBS/1%CAMHB (in order to avoid the re-activation of the metabolism in case of persister cells), as previously described (Grassi et al., 2017), and incubated at 37°C with and without 100x MIC of vancomycin. MRSA cells dislodged from an untreated biofilm were inoculated (final concentration ∼1 × 10^5^ CFUs/ml) either in PBS/1%CAMHB or in CAMHB and used as cell viability controls. After 1, 3 and 6 h-incubation, samples were serially diluted and plated on TSA for CFUs counting (plating detection limit = 50 CFUs/ml).

The time needed for persisters to revert into normal-growing cells was monitored by real-time investigation of heat production by IMC. Briefly, bacterial biofilm was formed and treated for 24 h as reported above. After sonication of beads, bacteria were diluted to ∼1 × 10^5^ CFUs/ml and incubated in glass ampoules filled with fresh CAMHB. Untreated biofilm was sonicated as well and diluted free-floating bacteria were incubated in CAMHB and used as control. Heat flow (μW) and total heat (J) were monitored for 15 h at 37°C.

### Antimicrobial Activity of Daptomycin Versus Persister Cells Survived After Vancomycin Treatment

To evaluate a potential synergistic eradicating activity of the sequential use of vancomycin and daptomycin against staphylococcal biofilm, a 24 h-old MRSA biofilm was formed on porous glass beads, as previously reported. After incubation, beads were rinsed (3x) and exposed to high vancomycin concentrations (1024 μg/ml) for 24 h and, after this time, beads were carefully rinsed and further exposed to sub-eradicating concentrations of daptomycin (16 μg/ml). After treatment suspension and sonication, serial dilutions of the sonication fluids were plated on TSA plates (plating detection limit = 50 CFUs/ml) and colonies were counted after 24 h incubation at 37°C.

### Data Analysis

Isothermal microcalorimetry data analysis was accomplished using the manufacturer’s software (TAM Assistant; TA Instruments, New Castle, DE, United States). The resulting data were expressed as heat flow (μW) versus time (h), as measure of the instantaneous heat produced at any time point, and as heat (J) versus time (h), as cumulative amount of heat produced during the experiment. Figures were plotted using GraphPad Prism 6.00 (GraphPad Software, La Jolla, CA, United States). From IMC data, the microbiologically relevant information growth rate (μ, J/h) and lag phase (λ, h) were derived according to growth models, as previously reported (Yang et al., 2007; Howell et al., 2011; Braissant et al., 2013).

## Results

### Antimicrobial Susceptibility Testing

Several antibiotics were tested against MRSA and MSSA. The respective MICs are reported in Table 1. Planktonic cells of both strains were susceptible to the assayed drugs by broth macrodilution, according to the EUCAST breakpoint recommendations (EUCAST, 2017). Rifampicin exhibited the lowest MIC = 0.008 μg/ml, followed by trimethoprim/sulfamethoxazole and daptomycin (MIC = 0.12 and 0.25 μg/ml, respectively). Vancomycin MIC (1 μg/ml) resulted slightly lower than that observed for fosfomycin and linezolid (both 2 μg/ml).

**Table 1:** The MIC_broth_, MIC_Etest_, MHIC and MBBC (μg/ml) of tested antibiotics against planktonic and biofilm MRSA and MSSA.

	MRSA				MSSA			
Antibiotic	MIC_broth_	MIC_Etest_	MHIC	MBBC	MIC_broth_	MIC_Etest_	MHIC	MBBC
DAP	0.25	0.25	0.25	32	0.25	0.5	0.25	32
FOS	2	1	2	>1024	2	0.75	2	>1024
VAN	1	1	2	>1024	1	1	1	>1024
TMP/SXT	0.25	n.t.	0.5	>256	0.12	n.t.	0.5	>256
LZD	2	n.t.	2	>1024	2	n.t.	4	>1024
RIF	0.008	0.006	0.016	128	0.016	0.012	0.008	256


*Daptomycin (DAP), fosfomycin (FOS), vancomycin (VAN), trimethoprim/sulfamethoxazole (TMP/SXT), linezolid (LNZ), and rifampicin (RIF). TMP/SXT concentrations are expressed as trimethoprim concentrations. n.t., not tested.*

No differences in MRSA and MSSA susceptibility profiles were observed, except when bacteria were challenged with trimethoprim/sulfamethoxazole and rifampicin. The former showed a twofold lower MIC against MSSA, while the latter a twofold higher MIC (0.12 and 0.016 μg/ml, respectively). These data were confirmed by Etest, which showed similar results for the antibiotics assayed within a twofold dilution range (Table 1).

### Evaluation of the Antibiotic Activity Against Planktonic Bacteria by IMC

Supporting Information Figure 1, 2 depict the heat flow (μW) curves generated by the metabolic activity of planktonic MRSA (Supplementary Figure S1) and MSSA (Supplementary Figure S2), when incubated with different concentrations of antimicrobials. The heat produced by the tested strains is reported instantaneously at any time point. The mathematical integration of this set of values is referred to as total heat (J), which represents the heat developed by the bacterial metabolic activity during the complete duration of the experimental observation (Supplementary Figure S1, S2). MHIC values determined by IMC ranged between 0.008 and 4 μg/ml (when testing rifampicin and linezolid, respectively), exhibiting a good correlation with data obtained using standard susceptibility testing (Table 1).

**FIGURE 1:**
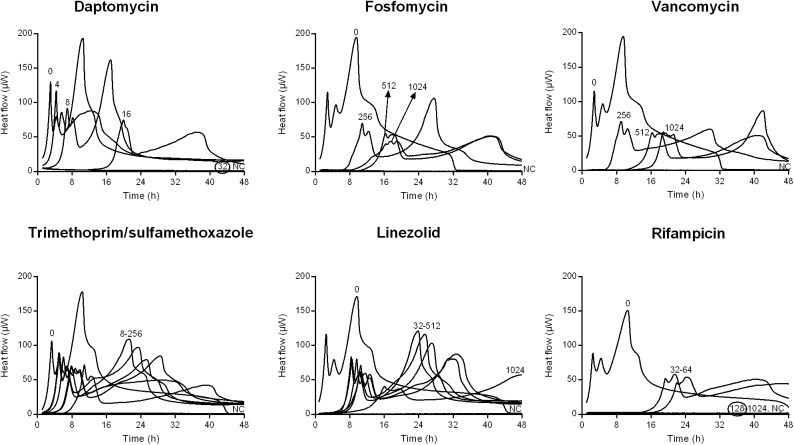
Isothermal microcalorimetry evaluation of antimicrobial activity of daptomycin, fosfomycin, vancomycin, trimethoprim/sulfamethoxazole, linezolid, and rifampicin against biofilm MRSA. Numbers represent antibiotic concentrations (μg/ml). A negative control has been used to confirm medium sterility (NC). Trimethoprim/sulfamethoxazole concentrations are expressed as the trimethoprim concentrations. Circled values represent the MBBC. Representative data of replicated experiments are reported.

**FIGURE 2:**
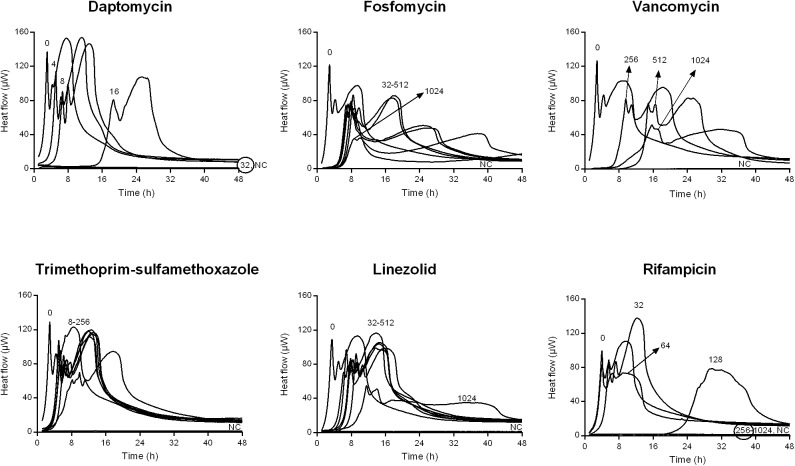
Isothermal microcalorimetry evaluation of antimicrobial activity of daptomycin, fosfomycin, vancomycin, trimethoprim/sulfamethoxazole, linezolid, and rifampicin against biofilm MSSA. Numbers represent antibiotic concentrations (μg/ml). A negative control has been used to confirm medium sterility (NC). Trimethoprim/sulfamethoxazole concentrations are expressed as the trimethoprim concentrations. Circled values represent the MBBC. Representative data of replicated experiments are reported.

### Evaluation of the Anti-Biofilm Activity by IMC and Sonication/Colony Counting

The activity of all antibiotics was also evaluated against MRSA and MSSA biofilms formed on porous glass beads and the viability of cells remaining on the bead was analyzed by IMC.

Figure 1, 2 shows the microcalorimetric curves generated by the heat flow related to the metabolic activity of biofilm cells still viable after the antibiofilm treatment (the corresponding total heat graphs are reported in Supplementary Figures S3, S4, respectively). The most effective biofilm bactericidal activity was exerted by daptomycin (MBBC: 32 μg/ml) and rifampicin (MBBC: 128–256 μg/ml) against both MRSA and MSSA strains. Conversely, a strong bactericidal effect was not detected when the staphylococcal biofilms were challenged with concentrations of fosfomycin, vancomycin, and linezolid up to 1024 μg/ml.

**FIGURE 3:**
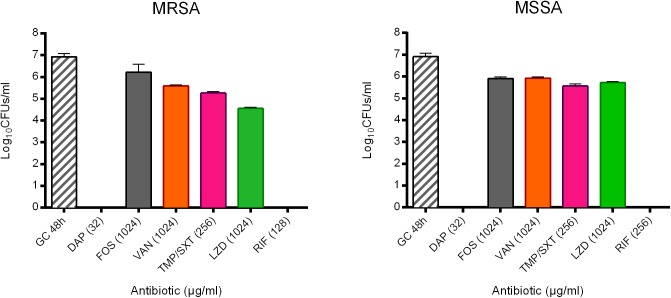
Evaluation of the anti-biofilm activity of the highest tested concentrations (μg/ml) of daptomycin (DAP), fosfomycin (FOS), vancomycin (VAN), trimethoprim/sulfamethoxazole (TMP/SXT), linezolid (LZD) and rifampicin (RIF) against biofilm of either MRSA or MSSA by sonication and colony counting (Log10 CFUs/mL). GC 48h; growth control of untreated 48 h-old biofilm. Data are expressed as mean ± SD, *n* = 3.

These observations are in agreement with the results obtained after sonication and colony counting (Figure 3). The number of cells from untreated biofilms used as positive control were 8.56 ± 3.59 × 10^6^ CFUs/ml and 8.13 ± 3.65 × 10^6^ CFUs for MRSA and MSSA, respectively. No colonies were detected after treating 24 h-old biofilms of both strains with either daptomycin or rifampicin, thus MBBCs for these antibiotics corresponded to the MBEC. When compared to the untreated biofilm, the highest tested concentrations of fosfomycin, vancomycin, and trimethoprim/sulfamethoxazole against biofilm MRSA determined a CFU number reduction of at least 1 log_10_, whereas linezolid reduced the staphylococcal biofilm more than 2 log_10_ CFU s/ml.

Conversely, for MSSA, a biofilm reduction exceeding 1 log_10_ was achieved when treating the biofilm with trimethoprim/sulfamethoxazole and linezolid. The treatment with fosfomycin and vancomycin resulted in a lower anti-biofilm activity, with a biofilm reduction remaining lower than 1 log_10_.

**FIGURE 4:**
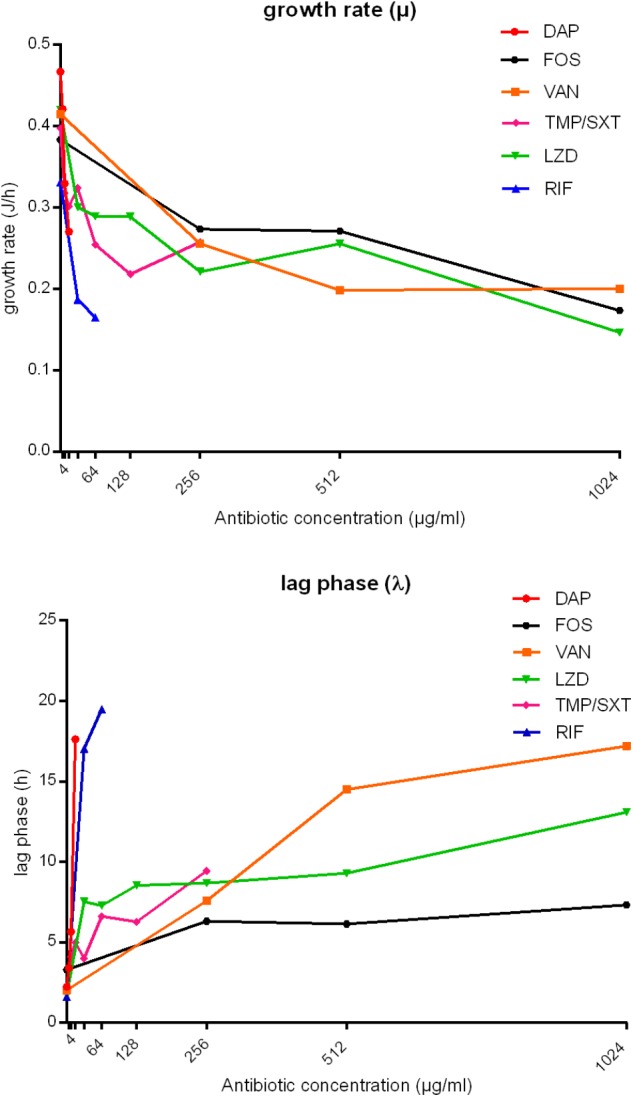
Analysis of growth rate (μ, J/h) and lag phase (λ, h) obtained from IMC data of MRSA biofilm treatment with daptomycin (DAP), fosfomycin (FOS), vancomycin (VAN), trimethoprim/sulfamethoxazole (TMP/SXT), linezolid (LNZ) and rifampicin (RIF). The bacterial growth rate (μ, J/h) and the lag phase (λ, h) are plotted against antibiotic concentrations (μg/ml). TMP/SXT concentrations are expressed as trimethoprim concentrations.

The timely anti-biofilm activity of daptomycin and rifampicin was further suggested by the mathematical analysis of IMC curves. Indeed, as shown for MRSA in Figure 4, a sharp decrease in the growth rate (μ (J/h)) was observed when biofilm was treated with increasing concentrations of these two antibiotics, up to the MBBCs, as compared to the untreated control. A reduction from ∼0.47 to ∼0.27 J/h was calculated already after treatment with 16 μg/ml daptomycin and a Δμ ∼-0.17 J/h (from ∼0.33 to ∼0.16 J/h) was observed when biofilm was challenged with 64 μg/ml rifampicin. Treatment with trimethoprim/sulfamethoxazole also induced a decrease in the metabolic functions of the challenged biofilm until a concentration of 128 μg/ml (Δμ ∼-0.18 J/h, calculated from μ of untreated and treated sample (128 μg/ml)). A doubled concentration (256 μg/ml) did not further affect the biofilm cells growth (Δμ ∼ 0.04 J/h). Linezolid and fosfomycin did not cause a similarly remarkable growth reduction after 24 h-treatment with increasing concentrations (Δμ ∼-0.05 J/h from 32 to 512 μg/ml and Δμ ∼-0.003 J/h from 256 to 512 μg/ml, respectively). Similarly, vancomycin highest tested concentrations (from 512 to 1024 μg/ml) did not deeply affect the bacterial metabolic growth pattern (Δμ ∼-0.05). A comparable anti-biofilm behavior could be appreciated when analyzing the lag phase [λ (h)] between the experiment start and the onset of the exponential growth. Indeed, the number of biofilm viable cells drastically decreased after being challenged with increasing, yet lower absolute concentrations of daptomycin and rifampicin, thus leading to a noticeable delay in the heat flow production (Δλ ∼ 15.38 h from 0 to 16 μg/ml and Δλ ∼ 17.89 h from 0 to 64 μg/ml, respectively). A gradual increase in λ was observed after trimethoprim/sulfamethoxazole treatment (Δλ ∼ 5.35 h from 8 to 256 μg/ml) and, in accordance with the growth rate data, fosfomycin (Δλ ∼ 1.01 h from 256 to 1024 μg/ml) and linezolid (Δλ ∼1.77 h from 32 to 512 μg/ml and Δλ ∼3.79 h from 512 to 1024 μg/ml) did not give a strong delay in the start of exponential growth phase using gradually increasing concentrations. High concentrations of vancomycin (512 and 1024 μg/ml) demonstrated to induce a similar time delay (Δλ ∼2.71 h), in accordance with the effect exerted on the growth rate. Similar results were also obtained from the analysis of the heat flow curves produced by MSSA biofilm after the treatment with the same antibiotics (Supplementary Figure S5).

Based on the analysis of the total heat production related to the biofilm recovery after 48 h-incubation in the microcalorimeter (Table 2), a minimum reduction of heat developed by biofilm samples of around 25% was estimated after treatment with concentrations ranging from 16 to 1024 μg/ml of the different tested antibiotics, as compared to the growth control. The only exception was recorded after MSSA antibiofilm treatment with trimethoprim/sulfamethoxazole and linezolid, which induced a total heat reduction not exceeding the threshold of 25%. To increase the reduction rate over 50% during 48 h-incubation, a minimum of twofold concentrated antibiotic dilutions were generally needed (≥32 μg/ml). Nevertheless, trimethoprim/sulfamethoxazole did not halve the metabolic activity of the treated samples in comparison to the untreated biofilm even at concentrations higher than 128 μg/ml. The same trend of increasing antibiotic concentrations was not observed when analyzing heat reductions exceeding 75%. Indeed, bactericidal compounds like daptomycin and rifampicin gave a heat reduction ≥ 75% with the same concentrations (MBBCs = 32, 128, and 256 μg/ml) that also decreased the total heat value over 48 h-analysis, whereas the other drugs did not succeed in strongly reducing the total amount of heat associated to the biofilm recovery after treatment.

**Table 2:** Percentage of reduction in heat (J) produced by MRSA and MSSA biofilms treated with the different tested antibiotics.

	MRSA			MSSA		
Antibiotic	≥25%	≥50%	≥75%	≥25%	≥50%	≥75%
DAP	16	**32**	**32**	**32**	**32**	**32**
FOS	256	–	–	1024	1024	–
VAN	256	1024	–	1024	–	–
TMP/SXT	128	–	–	–	–	–
LZD	32	1024	–	–	–	–
RIF	32	**128**	**128**	128	**256**	**256**


*Daptomycin (DAP), fosfomycin (FOS), vancomycin (VAN), trimethoprim/sulfamethoxazole (TMP/SXT), linezolid (LNZ), and rifampicin (RIF). Numbers in bold represent the MBBCs and concentrations are expressed in μg/ml. TMP/SXT concentrations are expressed as trimethoprim concentrations. n.t., not tested.*

### Isolation and Characterization of Persister Cells From MRSA Biofilm After Treatment With High Concentrations of Vancomycin

**FIGURE 5:**
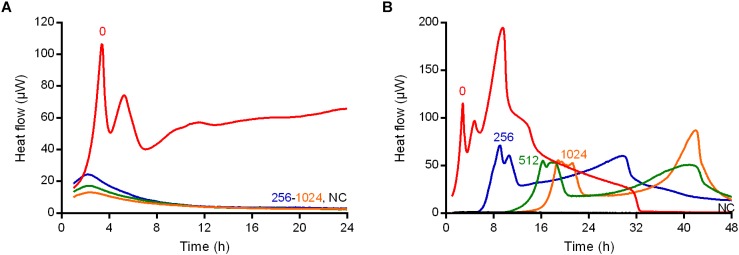
Isothermal microcalorimetry evaluation of anti-biofilm activity of vancomycin against biofilm MRSA in real-time **(A)** during and **(B)** after 24 h-treatment. Numbers represent antibiotic concentrations (μg/ml). A negative control has been used to confirm medium sterility (NC). Representative data of replicated experiments are reported.

The potential selection of persister cells in *S. aureus* biofilm due to the treatment with high bactericidal antibiotic concentrations (vancomycin) was also investigated. To do so, two different experiments using IMC were performed (Figure 5). As bacterial cells in a persister status are less metabolically active, they do not produce detectable heat (Grassi et al., 2017), but the heat can be measured again when cells revert to a normal-growing phenotype. First, the heat produced by 24 h-old biofilms of MRSA incubated with vancomycin was monitored in real time in the calorimeter (Figure 5A). After 24 h measurement beads were collected (washed to remove the antibiotic) and analyzed one more time by IMC for 48 h after inoculation in fresh medium (Figure 5B).

As observed during the IMC analysis of the MRSA biofilm treated with vancomycin (Figure 5A), all the drug concentrations (ranging from 256 to 1024 μg/ml) used determined a suppression of any detectable thermogenic process.

By contrast, the same samples after the vancomycin treatment and re-inoculation in fresh medium, produced heat (≈50 μW maximum peak), suggesting that some bacteria were still present on the beads after the 24 h-incubation (Figure 5B). However, a lower heat production and a delay in the time of the heat detection were observed in comparison to the heat produced by the positive control (without drug pre-treatment), suggesting that a less active metabolic status might characterize the biofilm cells after the treatment with vancomycin (Figure 5B).

**FIGURE 6:**
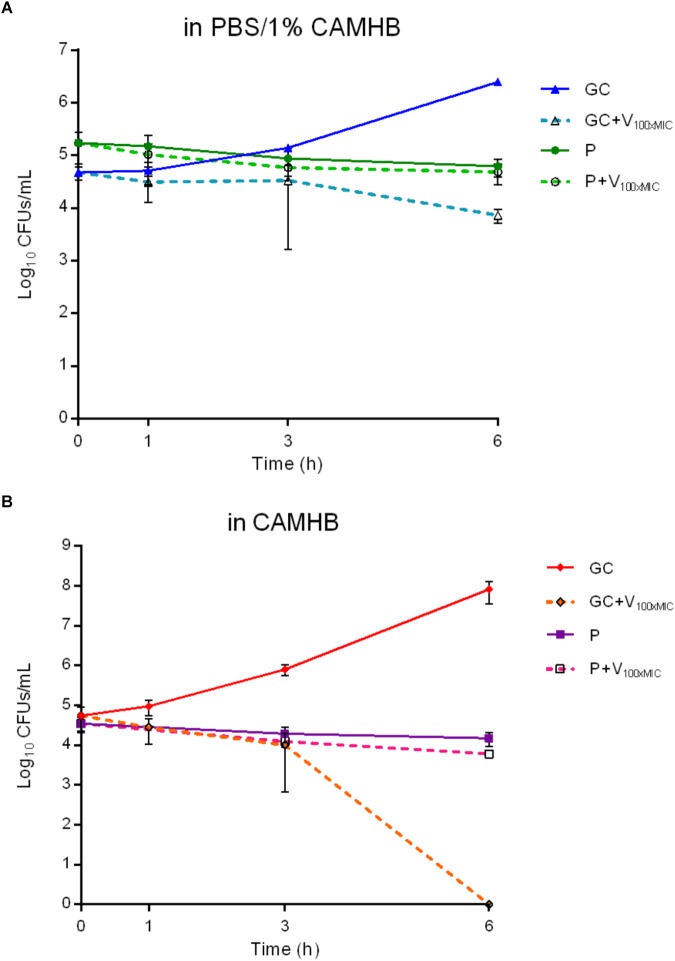
Characterization of persister cells isolated from MRSA biofilm after 24 h-treatment with 1024 μg/ml vancomycin. The exposure to high bactericidal concentrations of vancomycin (100x MIC_V AN_ = 100 μg/ml) was evaluated by colony counting (Log_10_CFUs/mL) of MRSA persister cells during 6 h-incubation in **(A)** PBS/1% CAMHB and **(B)** CAMHB. GC, growth control; GC+V_100xMIC_, growth control exposed to 100xMIC vancomycin; P, persister cells; P+V_100xMIC_ persister cells exposed to 100xMIC vancomycin. Data are expressed as mean ± SD, 3 ≤ n ≤ 6.

In order to evaluate the presence of cells in a persister status and exclude the selection of naturally occurring vancomycin-resistant mutants, MRSA biofilm cells treated with 1024 μg/ml drug were dislodged from glass beads and challenged with 100xMIC vancomycin (100 μg/ml) in PBS/1%CAMHB (Figure 6A). Suspensions were plated on solid medium at different time points. As depicted in Figure 6A, free-floating cells dislodged from treated biofilms and re-incubated with 100xMIC vancomycin (P+VAN_100xMIC_) seemed not to be affected by the high drug concentrations, as indicated by the CFUs number observed during 6 h-incubation. No difference in the CFUs number was observed for the same sample of bacteria derived from pre-treated biofilms (P), which were not challenged with 100xMIC vancomycin, in comparison to the value obtained for P+VAN_100xMIC_ sample. By contrast, cells derived from biofilm without any drug pre-treatment (GC) showed an increase of 1.5 log_10_ CFU/ml in comparison to the initial inoculum after 6 h-incubation in PBS+1%CAMHB. The same samples exposed to 100xMIC vancomycin (GC+VAN_100xMIC_) showed a reduction of ∼ 2.5 log_10_ CFU/ml after 6 h in comparison to the untreated GC (7.2 ± 2.1 × 10^3^ vs. 2.4 ± 3.9 × 10^6^ CFUs/ml respectively), indicating a higher susceptibility to 100xMIC vancomycin than that observed P+VAN_100xMIC_ sample.

In order to confirm the bactericidal activity of vancomycin used in the experiment, the antibiotic was tested against free-floating bacteria (dislodged from untreated biofilm) in fresh medium (Figure 6B). Cells incubated in broth reached an early exponential growth within 6 h (from 5.5 ± 3.2 × 10^4^ to 8.2 ± 4.6 × 10^7^ CFUs/ml), whereas, when exposed to 100xMIC vancomycin, a complete killing of free-floating bacteria was observed after 6 h-incubation.

**FIGURE 7:**
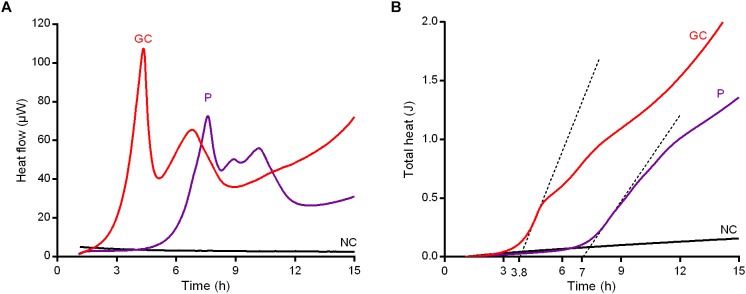
Characterization of persister cells isolated from MRSA biofilm after 24 h-treatment with 1024 μg/ml vancomycin. Revival assay in CAMHB by IMC monitoring **(A)** the heat flow (μW) plotted against time (h) and **(B)** the total heat (J) produced during the experimental time (h). GC, growth control; P, persister cells; NC, negative control. Representative data of replicated experiment are reported.

To further confirm our hypothesis that the anti-biofilm treatment with vancomycin induced persistence in MRSA biofilm, treated biofilm cells were re-incubated in rich growth medium after sonication to evaluate by IMC the characteristic delay in heat production (lag phase λ) in comparison to an untreated control, due to the reversion to a normal growing phenotype already observed by Grassi et al. (2017). As depicted in Figure 7A, both GC and P samples produced heat in fresh medium (with a trend similar to the curves depicted in Figure 5B). However, a clear temporal shift was observed in heat flow curves produced by P compared to untreated GC. Indeed, a Δλ = 3.2 h was calculated between the first peaks of GC (λ = 3.8 h) and P (λ = 7 h) (Figure 7B), suggesting that the cells of sample P remained in a metabolically inactive status and, after 6 h (the time needed for the treated sample’s heat flow to exceed the detection limit of ∼10^4^–10^5^ CFUs/ml (Butini et al., 2018b) incubation in fresh CAMHB), they reverted into metabolically active cells (Supplemetary Figure S6).

### Bactericidal Effect of Vancomycin and Daptomycin Staggered Administration Against MRSA Biofilm

**FIGURE 8:**
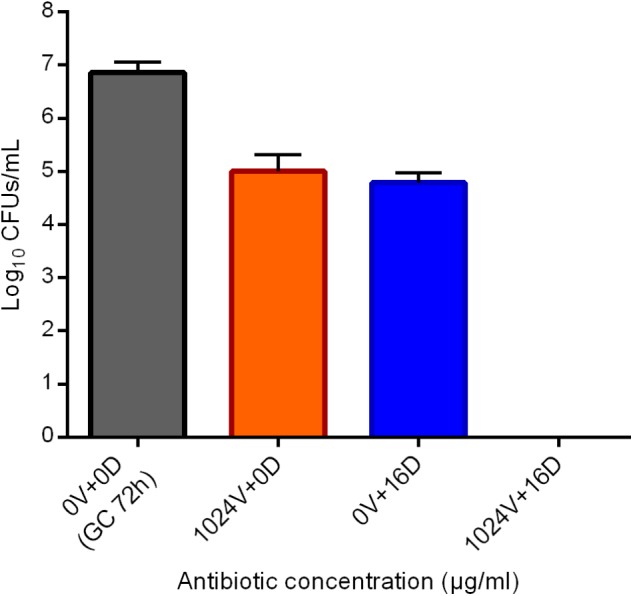
Evaluation of the eradicating activity of the sequential treatment vancomycin/daptomycin against a 24 h-old MRSA biofilm by sonication and colony counting (Log_10_CFUs/mL). Numbers indicate vancomycin (V) and daptomycin (D) concentrations (μg/ml). GC 72h; growth control of untreated 72 h-old biofilm. Data are expressed as mean ± SD, *n* = 3.

Based on the assumption that vancomycin efficiently kills the susceptible portion of the staphylococcal biofilm community, thus selecting for persistent cells, a possible synergistic eradicating activity of a sequential administration of bactericidal concentration of vancomycin (1024 μg/ml) and sub-eradicating concentration of daptomycin (16 μg/ml) was evaluated. As depicted in Figure 8, the bacterial load of a 24 h-old biofilm firstly treated with 1024 μg/ml vancomycin and then receiving no daptomycin was reduced by ∼2 log_10_. Similarly, a first 24 h-incubation without vancomycin treatment and a following exposition to 16 μg/ml daptomycin resulted in a ∼2 log_10_. Interestingly, when sequentially combined, a 24 h-treatment with vancomycin, followed by 24 h-incubation with daptomycin, showed a complete killing of biofilm viable cells, highlighting a synergistic eradicating activity against MRSA biofilm mostly populated by persister cells.

## Discussion

The determination of antimicrobial susceptibilities is of fundamental importance during the developmental procedure of new antimicrobial drugs’ discovery, as well as in predicting possible therapeutic outcomes during the pharmacological management of an infection. As around 80% of human infections are due to sessile bacteria, the development of biofilm-specific antimicrobial assays is needed (Percival et al., 2015; Ciofu et al., 2017).

Isothermal microcalorimetry is a highly sensitive technique that enables a real-time monitoring of bacterial viability in terms of metabolism-related heat production (Braissant et al., 2010). This method was widely used and validated in our laboratory for testing the anti-biofilm activity of different antimicrobial formulations, including antibiotics (Oliva et al., 2014; Butini et al., 2018a,b; Casadidio et al., 2018), antifungals (Furustrand Tafin et al., 2013), and bacteriophages (Tkhilaishvili et al., 2018a,b).

Here, the analysis of the activity of commonly prescribed antibiotics against two laboratory strains of *S. aureus* performed by IMC showed that both planktonic MSSA and MRSA were susceptible to all the tested antibiotics tested (EUCAST, 2017), but considerable higher concentrations; 128xMHIC and ≥ 8000xMHIC for daptomycin and rifampicin, respectively, were needed to eradicate the biofilm of the same reference strains. Moreover, it was not possible to determine the MBEC for the other antibiotics tested, as live bacterial cells were still attached to glass beads after treatment with the highest tested antibiotic concentrations. The tolerance of *S. aureus* biofilms (including those formed by clinical isolates) to high concentrations of different antibiotics was already observed *in vitro* (Singh et al., 2017; Di Luca et al., 2018) and *in vivo* (Patel et al., 2000) and it might be responsible for the failure of the monotherapy in the treatment of biofilm-associated infections (Jacqueline and Caillon, 2014).

In the past, the presence of the extracellular matrix has been proposed as responsible for the reduced permeability of the biofilm to antibiotics (Mathur et al., 2005). However, recent *in vitro* studies have showed that most antibiotics can penetrate biofilm quite efficiently (Stewart et al., 2009; Singh et al., 2010). Therefore, the presence of a subpopulation of cells (within the sessile community) extremely tolerant to conventional antibiotics could explain the difficulty in eradicating biofilms and the relapse of the infection after the antibiotic treatment (Conlon, 2014).

Interestingly, the microcalorimetric heat flow curves showed a delay in the heat detection and a different curve shape in comparison to the untreated biofilm control. Also the analysis of λ and μ indicated that, as compared to the untreated growth control, biofilm bacteria pre-treated with 1024 μg/ml vancomycin exhibited a much longer lag phase and a slower growth rate. This prolongation in lag time might be due to a lower number of bacteria on the beads given by the partial killing of the initial inoculum (Astasov-Frauenhoffer et al., 2014) after the antibiotic treatment, which also induced a clear decrease in bacterial metabolic activity, in comparison to the number and the metabolic status of the untreated control cells. These are recognized as characteristic features of persister cells isolated after antibiotic treatment. Moreover, specifically in case of vancomycin treatment, there is similarity between the growth rate/concentration curve (J/h/μg/ml) reported in Figure 4 and the persisters typical biphasic kill curve (bacterial inoculum/time, Log CFUs/ml/h) (Fisher et al., 2017).

To confirm whether the heat produced by the pre-treated biofilms (recorded by IMC) correlated with the presence of persister cells selected by the killing of normal-growing cells with the highest antibiotic concentrations, we focused our research on the effect of vancomycin versus an *in vitro* 24 h-old biofilm of MRSA.

When the effect of high concentrations of glycopeptide (ranging from 128 to 1024 μg/ml) was tested against the staphylococcal biofilm and monitored in real-time (co-incubation of vancomycin with biofilm in the microcalorimetric glass ampoules), no metabolism-related heat flow was detected (Figure 5A). By contrast, when the pre-treated biofilm samples were washed and re-incubated in a fresh medium without any antibiotic, the presence of a residual inoculum (∼5% as compared to the untreated biofilm population) on the beads produced a detectable amount of heat, which was observed for all the tested samples. Since this effect was similar to the reversion pattern to a normal-growing phenotype that was recently described by Grassi et al. (2017), it was reasoned that the bacterial cells selected after biofilm treatment with 1024 μg/ml vancomycin might be considered persister ones. The high tolerance of free-floating cells dislodged from MRSA biofilm pre-treated with vancomycin and subsequently challenged with 100xMIC of the same glycopeptide in PBS/1%CAMHB confirmed this hypothesis. Biofilm dislodged cells pre-treated with 1024 μg/ml vancomycin were not able to replicate (within 6 h) when maintained in a low-nutrient conditions and, once challenged with extremely high doses of vancomycin, the CFUs number remained rather constant over the time without any reduction. Therefore, these biofilm-dislodged cells might be considered persister. Nevertheless, when persisters were inoculated in fresh rich medium and analyzed once more by IMC, they re-activated their metabolic activities producing a detectable heat and, if then challenged with 100xMIC vancomycin, they could be killed, indicating a complete reversion to a normal-growing phenotype. In addition, these data also excluded the presence of vancomycin resistant clones.

We demonstrate that a deeper analysis of IMC curves allows to detect and recognize in real-time the presence of persister cells in a biofilm after receiving an anti-biofilm treatment, thus making this technique also suitable for the analysis and characterization of new anti-persisters molecules. Moreover, it was shown that the scarce efficacy of vancomycin administered alone against a 24 h-old *S. aureus* biofilm *in vitro* is mainly due to the presence/selection of persister cells, rather than resistance development or inadequate antibiotic penetration (Conlon, 2014). This might then explain the higher rate of failure of the treatment based only on the use of this glycopeptide in the clinical practice, if rifampin is not added in combination (Niska et al., 2013).

Interestingly, the tolerance of *S. aureus* to 100xMIC daptomycin has been also described in stationary phase cell population (Lechner et al., 2012). However, here we report that a sub-eradicating concentration of daptomycin (16 μg/ml), which determined a reduction of barely 2 log_10_ when tested alone against MRSA biofilm, was able to kill persisters cells remaining within the biofilm on the beads after vancomycin treatment. This observation suggests that the tolerance developed to vancomycin cannot be considered multi-drug like in the case of persisters raised during the inhibition of the translation (Keren et al., 2004).

Since vancomycin clearly killed susceptible cells and selected for non-growing dormant ones, we propose a sequential administration of a combined vancomycin/daptomycin treatment to achieve the killing of all viable biofilm cells. The present results support the use of daptomycin in the clinical practice against MRSA infection in case of failure with vancomycin therapy.

## Conclusion

We showed the selection/isolation of *S. aureus* persister cells from a 24 h-old biofilm after treatment with high concentrations of vancomycin. Moreover, IMC detecting in real-time the presence of these slowly-growing cells, which resuscitate upon treatment discontinuation, might prove to be a useful technique for the development and the investigation of anti-persister drugs.

## Data Availability

The datasets generated for this study are available on request to the corresponding author.

## Author Contributions

MB, AT, and ML conceived the study. MB and ML designed the experiments. MB performed the experiments. GA and CR performed the mathematical analysis of calorimetric curves. MB and ML analyzed data with the contribution of GA and CR. MB and ML drafted the manuscript, with the contribution of AT and GA.

## Conflict of Interest Statement

The authors declare that the research was conducted in the absence of any commercial or financial relationships that could be construed as a potential conflict of interest.
