# Supplementary material for: Isothermal Microcalorimetry Detects the Presence of Persister Cells in a Staphylococcus aureus Biofilm After Vancomycin Treatment
Source: Front Microbiol. 2019 Feb 25;10:332. doi: 10.3389/fmicb.2019.00332 (PMC6398423; doi:10.3389/fmicb.2019.00332)
Supplement: Supplementary file 1 [file Data_Sheet_1.pdf]

SUPPLEMENTARY INFORMATION

Isothermal microcalorimetry detects the presence of persister cells in a *Staphylococcus aureus* biofilm after vancomycin treatment

Maria Eugenia Butini[a,b], Gerardo Abbandonato[c], Carmine Di Rienzo[c,d], Andrej Trampuz[a,b] and Mariagrazia Di Luca[a,b,c, e@]

[a]Charité – Universitätsmedizin Berlin, corporate member of Freie Universität Berlin, Humboldt-Universität zu Berlin, and Berlin Institute of Health, Center for Musculoskeletal Surgery; Charitéplatz 1, 10117 Berlin, Germany

[b]Berlin-Brandenburg Center for Regenerative Therapies, Charité – Universitätsmedizin Berlin; Augustenburger Platz 1, 13353 Berlin, Germany

[c]NEST, Istituto Nanoscienze - Consiglio Nazionale delle Ricerche; Piazza San Silvestro 12, 10027 Pisa, Italy

[d]Center for Nanotechnology Innovation @ NEST, Istituto Italiano di Tecnologia, Pisa, Italy.

[e]Department of Biology, University of Pisa, via San Zeno 35-39, 56127 Pisa Italy

[@]**Corresponding author:** Dr. Mariagrazia Di Luca

Berlin-Brandenburg Center for Regenerative Therapies (BCRT)
Charité – Universitätsmedizin Berlin
Campus Virchow Klinikum
Augustenburger Platz 1 (Südstraße 2)
D-13353 Berlin
Germany
Email: diluca.mariagrazia@gmail.com

Running title: isothermal microcalorimetry reveals persister cells

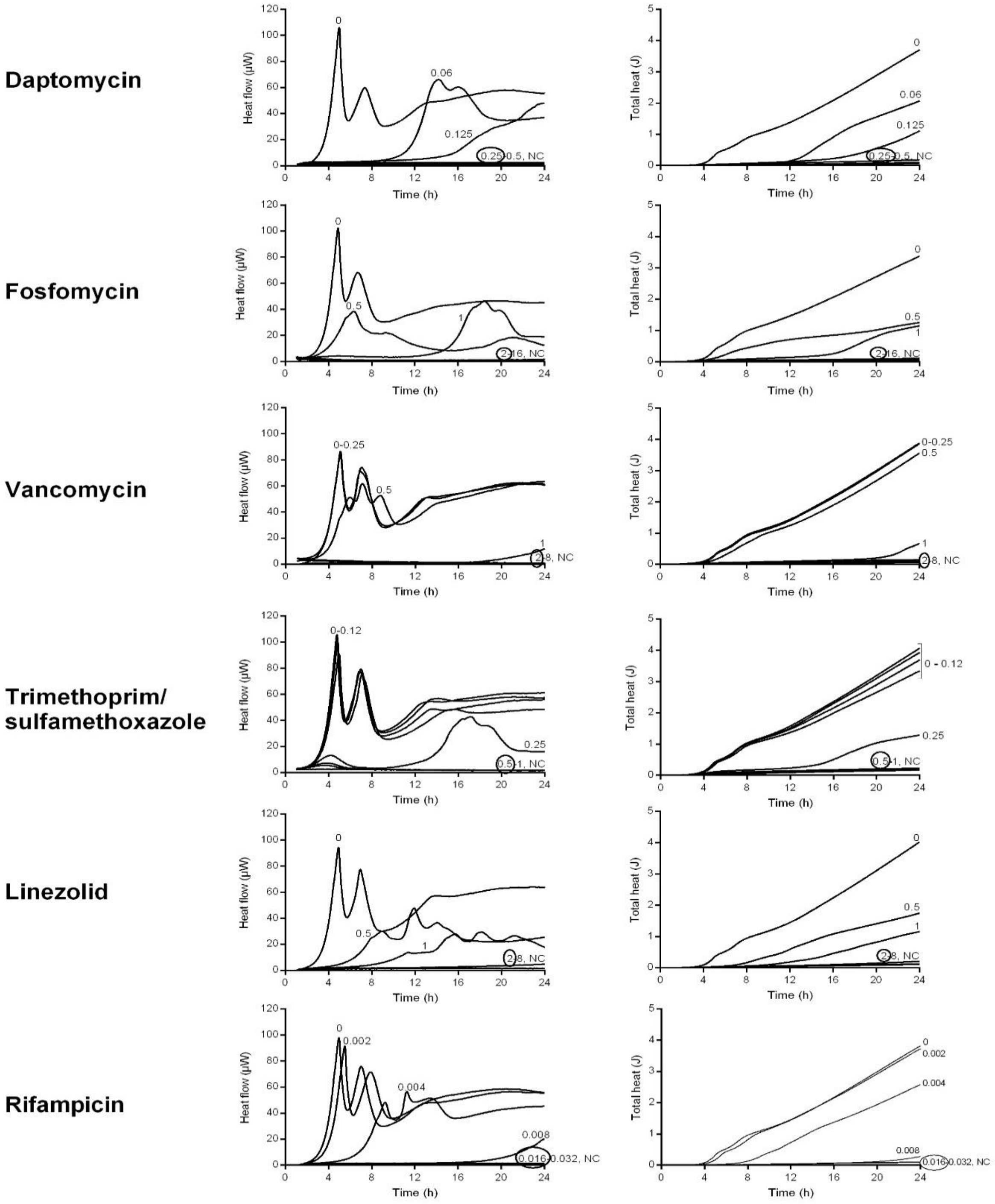

Figure S1 Evaluation of antimicrobial activity of daptomycin, fosfomycin, vancomycin, trimethoprim/sulfamethoxazole, linezolid and rifampicin against planktonic MRSA by IMC. Numbers represent antibiotic concentrations (µg/ml). Heat flow (µW) and total heat (J) are plotted against time (h). A negative control has been used to confirm medium sterility (NC). Trimethoprim/sulfamethoxazole concentrations are expressed as the trimethoprim concentrations. Circled values represent the MHIC. Representative data of replicated experiments are reported.

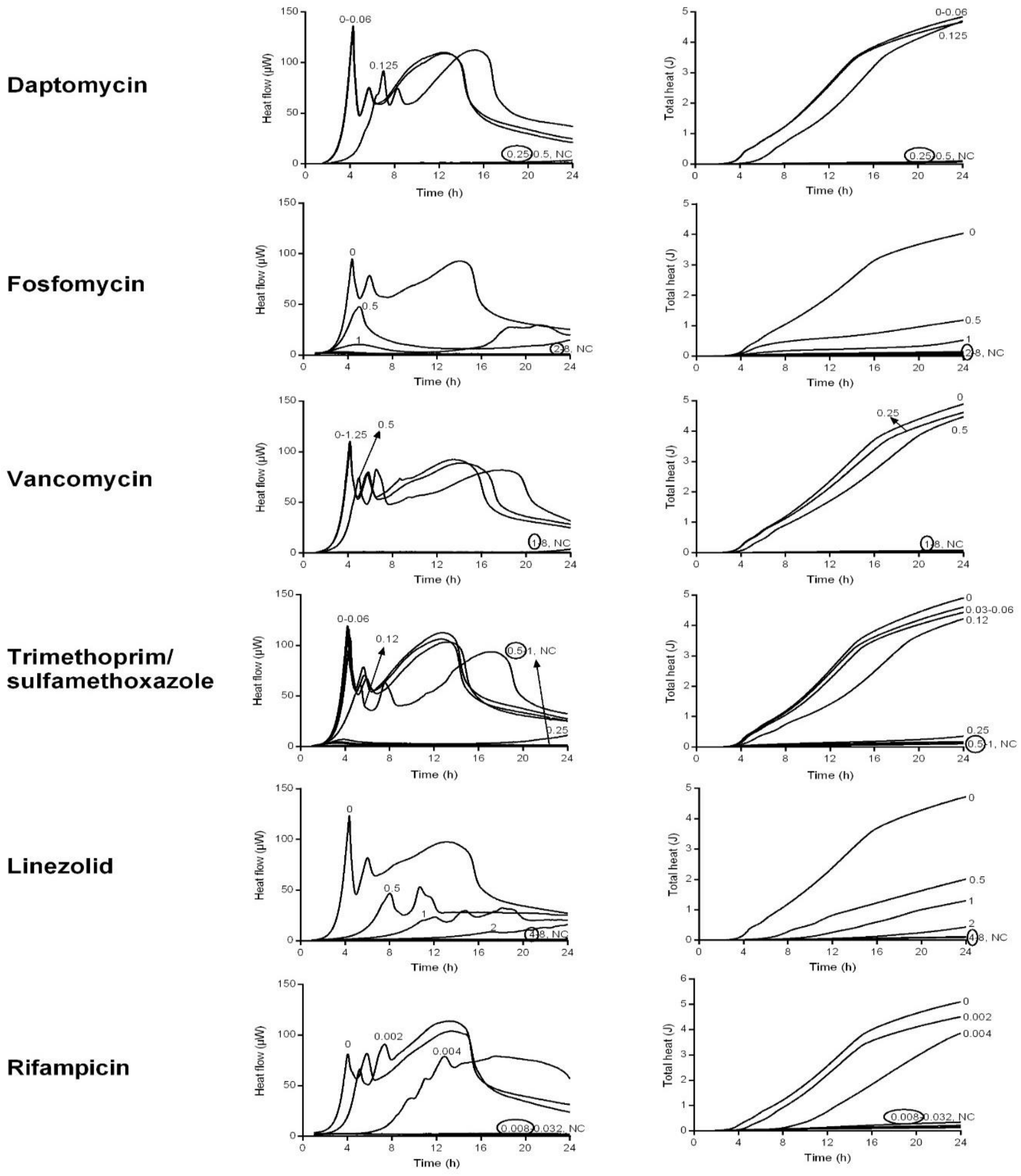

Figure S2 IMC evaluation of antimicrobial activity of daptomycin (A), fosfomycin (B), vancomycin (C), trimethoprim/sulfamethoxazole (D), linezolid (E) and rifampicin (F) against planktonic MSSA by IMC. Numbers represent antibiotic concentrations (µg /ml). Heat flow (µW) and total heat (J) are plotted against time (h). A negative control has been used to confirm medium sterility (NC). Trimethoprim/sulfamethoxazole concentrations are expressed as the trimethoprim concentrations. Circled values represent the MHIC. Representative data of replicated experiments are reported.

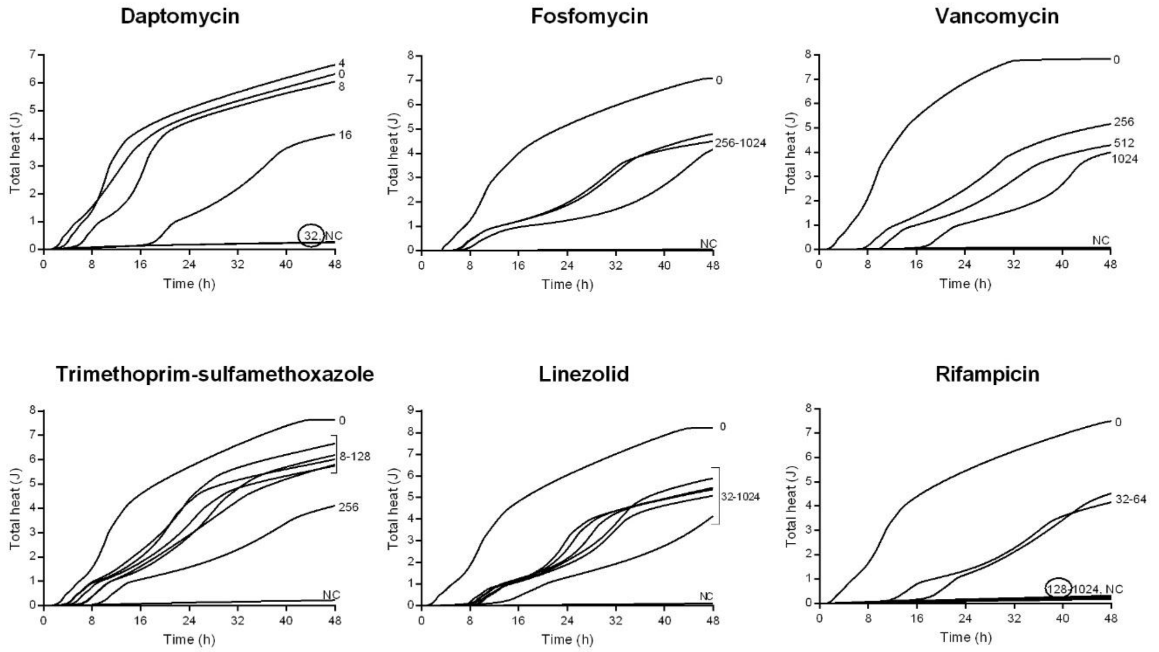

Figure S3 IMC evaluation of antimicrobial activity of daptomycin, fosfomycin, vancomycin, trimethoprim/sulfamethoxazole, linezolid and rifampicin against biofilm MRSA by microcalorimetry. Numbers represent antibiotic concentrations (µg/ml). Total heat (J) are plotted against time (h). A negative control has been used to confirm medium sterility (NC). Trimethoprim/sulfamethoxazole concentrations are expressed as the trimethoprim concentrations. Circled values represent the MBBC. Representative data of replicated experiments are reported.

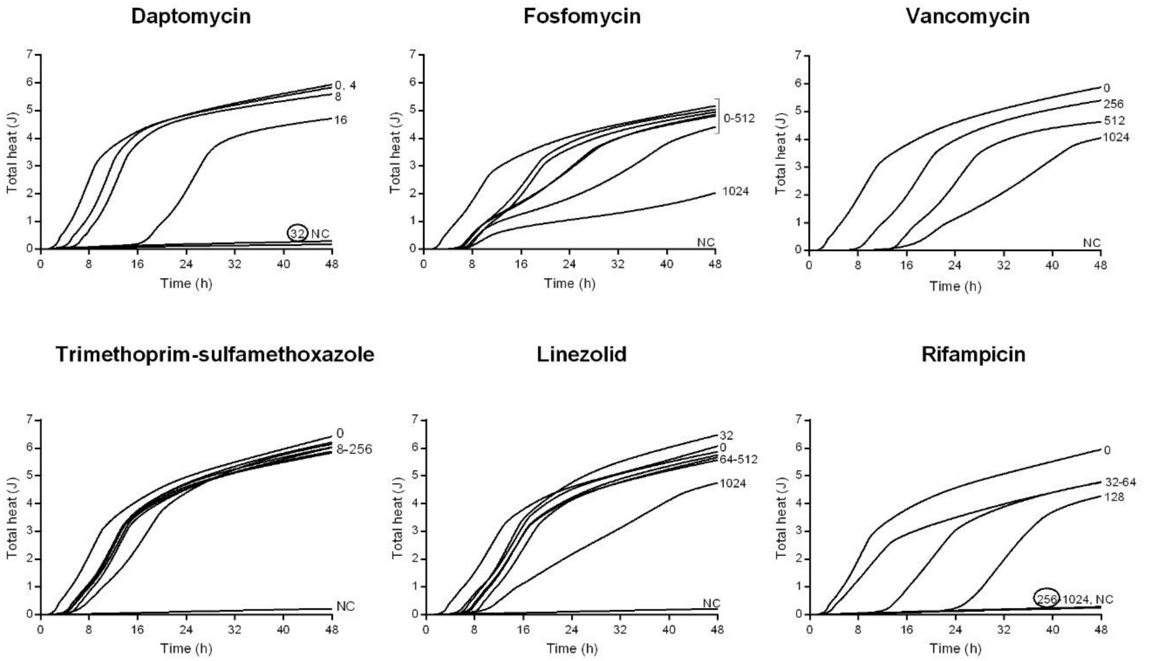

Figure S4 Evaluation of antimicrobial activity of daptomycin, fosfomycin, vancomycin, trimethoprim/sulfamethoxazole, linezolid and rifampicin against biofilm MSSA by microcalorimetry. Numbers represent antibiotic concentrations (μg /ml). Total heat (J) are plotted against time (h). A negative control has been used to confirm medium sterility (NC). Trimethoprim/sulfamethoxazole concentrations are expressed as the trimethoprim concentrations. Circled values represent the MBBC. Representative data of replicated experiments are reported.

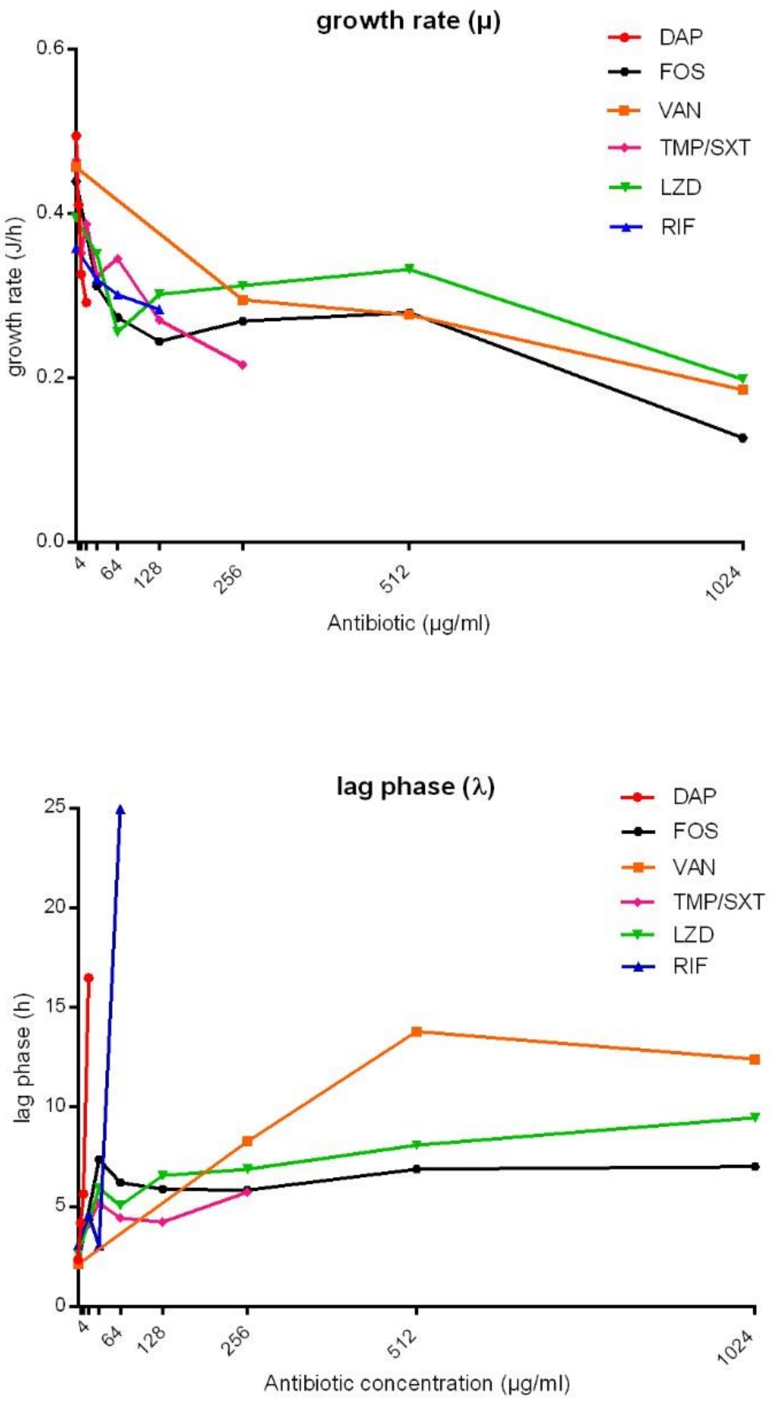

Figure S5 Analysis of growth rate (μ, J/h) and lag phase (λ, h) obtained from IMC data of MSSA biofilm treatment with daptomycin (DAP), fosfomycin (FOS), vancomycin (VAN), trimethoprim/sulfamethoxazole (TMP/SXT), linezolid (LNZ) and rifampicin (RIF). The bacterial growth rate (k, J/h) and the lag phase (λ, h) are plotted against antibiotic concentrations ranging from 4 to 1024 μg/ml. TMP/SXT concentrations are expressed as trimethoprim concentrations.

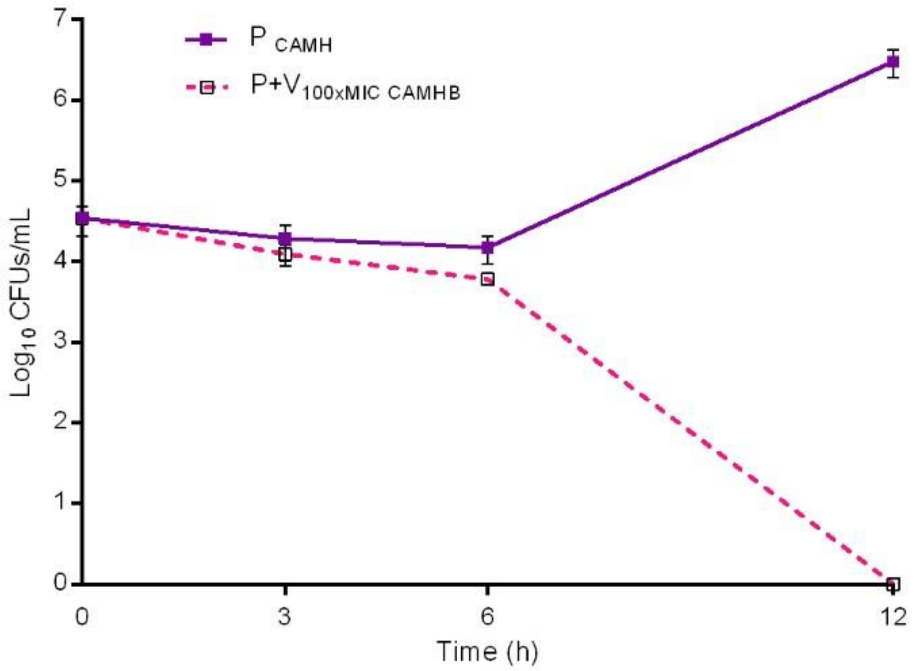

Figure S6 Characterization of persister cells (P) isolated from MRSA biofilm after 24h-treatment with 1024 µg/ml vancomycin. The exposure to high bactericidal vancomycin concentrations (100x MIC_{VAN} = 100 µg/ml) was evaluated by colony counting of MRSA persister cells (Log_{10}CFUs/mL) during 12h-exposure to 100 µg/mL vancomycin in CAMHB. P, persister cells in CAMHB without vancomycin; $P+V_{100xMIC\ CAMHB}$; persister cells in CAMHB with 100xMIC vancomycin. Data are expressed as mean ± SD, n=3.
